# Editorial: Multimodal treatment of recurrence and distant metastases of colorectal cancer

**DOI:** 10.3389/fonc.2024.1455405

**Published:** 2024-07-11

**Authors:** Andrea Balla, Diletta Corallino, Diego Coletta, Pierpaolo Sileri, Salomone Di Saverio, Salvador Morales-Conde

**Affiliations:** ^1^ Department of General and Digestive Surgery, University Hospital Virgen Macarena, University of Sevilla, Sevilla, Spain; ^2^ Unit of General and Digestive Surgery, Hospital Quirónsalud Sagrado Corazón, Sevilla, Spain; ^3^ Department of General Surgery and Surgical Specialties “Paride Stefanini”, Sapienza University of Rome, Rome, Italy; ^4^ Hepatobiliary Surgery Division, Istituto di Ricovero e Cura a Carattere Scientifico (IRCCS) San Raffaele Scientific Institute, Milan, Italy; ^5^ Hepatopancreatobiliary Surgery, IRCCS Regina Elena National Cancer Institute, Rome, Italy; ^6^ Colorectal Surgery Unit, IRCCS San Raffaele Scientific Institute, Vita-Salute University, Milan, Italy; ^7^ Azienda Sanitaria Unica Regionale (ASUR) Marche 5, San Benedetto del Tronto General Hospital, San Benedetto del Tronto, Italy

**Keywords:** rectal cancer (RC), chemotherapy, radiotherapy, local recurrence, distant metastases

For the treatment of rectal cancer and recurrence or distant metastases several critical improvements have been developed in the last decades. Since the first milestone intervention proposed by Miles more than one hundred years ago, for the treatment of low rectal tumors, surgery has made great progress with the introduction of low anterior resection with Knight and Griffits anastomosis, colonic pouches, intersphincteric resection, the introduction of the minimally invasive approach by laparoscopy or by robot, the use of transanal device, or the use of fluorescence angiography and lymphangiography, up to the use of artificial intelligence intraoperatively (1–3). However, we must not forget maybe the most important advance in the treatment of rectal cancer such as the description by Heald in the 80’s of the concept of total mesorectal excision (TME) (4). In fact the introduction of this new paradigm was a sliding door for the oncological results allowing to reduce drastically the recurrence rate after surgery (4). Similarly the introduction of the neoadjuvant chemo-radiotherapy (n-CRT) contributed to reduce dramatically the recurrence rate (4).

Anyway, rectal cancer, apart from local recurrence, is also responsible for distant metastases, especially liver metastases, and in the last years a great effort has been made to investigate about the tumor biology, in order to improve survival and disease free survival of these patients. I want to thank to Frontiers in Oncology to have the opportunity to serve as Editor of this monographic issue about the multimodal treatment of recurrence and distant metastases of colorectal cancer, and I want to thank all authors involved.

Important findings are reported in this Research Topic by using nomograms and machine learning, about the prediction of the survival outcomes for patients affected by young-onset colorectal cancer with the aim to assist in developing clinical treatment strategies for these patients (Li et al.), and about the prediction of distant metastatic sites and risk facilitating the clinical decision-making process (He et al. and Qiu et al., respectively).

On the other hand, Dai et al., He et al., Gao et al., and Zhou et al. focused their findings on the use of new protocol of immunotherapy, chemotherapy and radiotherapy for the treatment of metastatic colorectal cancer with encouraging results.

Li et al. reported an interesting literature review about acupuncture showing its use for the treatment of colorectal cancer symptoms, while Xu et al. reported as positive clinical circumferential resection margin is an independent risk factor for recurrence after TME. Finally, Baba et al. reported as irinotecan induced interstitial lung disease even in a patients underwent bone marrow transplantation for aplastic anemia decades before.

Treatment of rectal cancer, especially in case of local recurrence or distant metastases is still a debated topic and further topics will be of interest in the future, however, we considered that the present issue includes high-quality studies. We hope that this monographic issue will be of interest for the reader, helping to update the most advanced knowledge on rectal cancer treatment.

## Author contributions

AB: Conceptualization, Data curation, Formal analysis, Funding acquisition, Investigation, Methodology, Project administration, Resources, Software, Supervision, Validation, Visualization, Writing – original draft, Writing – review & editing. DCor: Conceptualization, Data curation, Formal analysis, Funding acquisition, Investigation, Methodology, Project administration, Resources, Software, Supervision, Validation, Visualization, Writing – original draft, Writing – review & editing. DCol: Conceptualization, Data curation, Formal analysis, Funding acquisition, Investigation, Methodology, Project administration, Resources, Software, Supervision, Validation, Visualization, Writing – original draft, Writing – review & editing. PS: Conceptualization, Data curation, Formal analysis, Funding acquisition, Investigation, Methodology, Project administration, Resources, Software, Supervision, Validation, Visualization, Writing – original draft, Writing – review & editing. SDS: Funding acquisition, Investigation, Methodology, Project administration, Resources, Software, Supervision, Validation, Visualization, Writing – original draft, Writing – review & editing, Conceptualization, Data curation, Formal analysis. SM-C: Conceptualization, Data curation, Formal analysis, Funding acquisition, Investigation, Methodology, Project administration, Resources, Software, Supervision, Validation, Visualization, Writing – original draft, Writing – review & editing.
